# Transcriptomic Profile of Primary Culture of Skeletal Muscle Cells Isolated from Semitendinosus Muscle of Beef and Dairy Bulls

**DOI:** 10.3390/ijms21134794

**Published:** 2020-07-07

**Authors:** Anna Ciecierska, Tomasz Motyl, Tomasz Sadkowski

**Affiliations:** 1 Department of Human Nutrition, Institute of Human Nutrition Sciences, Warsaw University of Life Sciences - SGGW, Nowoursynowska 159C, 02-776 Warsaw, Poland; anna_ciecierska@sggw.edu.pl; 2 Department of Physiological Sciences, Institute of Veterinary Medicine, Warsaw University of Life Sciences - SGGW, Nowoursynowska 159, 02-776 Warsaw, Poland; tomasz_motyl@sggw.edu.pl

**Keywords:** cattle, gene expression, microarray, myogenesis, satellite cells, skeletal muscle

## Abstract

The aim of the study was to identify differences in the transcriptomic profiles of primary muscle cell cultures derived from the semitendinosus muscle of bulls of beef breeds (Limousin (LIM) and Hereford (HER)) and a dairy breed (Holstein-Friesian (HF)) (*n* = 4 for each breed). Finding a common expression pattern for proliferating cells may point to such an early orientation of the cattle beef phenotype at the transcriptome level of unfused myogenic cells. To check this hypothesis, microarray analyses were performed. The analysis revealed 825 upregulated and 1300 downregulated transcripts similar in both beef breeds (LIM and HER) and significantly different when compared with the dairy breed (HF) used as a reference. Ontological analyses showed that the largest group of genes were involved in muscle organ development. Muscle cells of beef breeds showed higher expression of genes involved in myogenesis (including *erbb-3*, *myf5*, *myog*, *des*, *igf-1*, *tgfb2*) and those encoding proteins comprising the contractile apparatus (*acta1*, *actc1*, *myh3*, *myh11*, *myl1*, *myl2*, *myl4*, *tpm1*, *tnnt2*, *tnnc1*). The obtained results confirmed our hypothesis that the expression profile of several groups of genes is common in beef breeds at the level of proliferating satellite cells but differs from that observed in typical dairy breeds.

## 1. Introduction

It is known that different cattle breeds are characterized by a different structure and physiology of skeletal muscles. Beef breeds such as the late-maturing Limousin (LIM) and the early-maturing Hereford (HER) are expected to express differences in the amount of muscle and adipose tissue in the carcass relative to the typical dairy breed such as the Holstein-Friesian (HF). It still remains unknown as to which genes determine the interracial differences in the rate of growth and metabolism of the muscle tissue [1].

Literature data indicate that the ratio of skeletal muscles to the weight of carcass increases in the early period of bulls’ life and decreases from the sixth month due to additional deposition of the adipose tissue and connective tissue. However, around a 15-fold increase in the muscle mass occurs in this period [1,2]. It has been demonstrated that between 180 and 300 days of life the highest daily muscle tissue growth is observed, which is approximately 400 g per day, followed by a decrease. In addition, in the same period, a slow increase in the mass of adipose tissue is observed, which is approximately 100 g per day [2,3].

Satellite cells are small, undifferentiated, mononuclear precursor cells of the skeletal muscles found in adult individuals and involved in adaptive processes of growth, repair, and regeneration of the muscle tissue in the postnatal period [4,5,6]. Under physiological conditions, the satellite cells present in mature skeletal muscles remain quiescent until their activation (e.g., via muscle tissue injury) and constitute a pool of primary cells enabling the repair and regeneration of damaged muscle fibers as well as increasing muscle mass in the postnatal period [7,8,9]. In the postnatal period, the number of muscle fibers remains unchanged, while an intensive growth of muscle mass occurs as a result of fusion of satellite cells located directly at the growing muscles fibers. It has been determined that the majority of cell nuclei in muscle fibers of adult mammals originate from myoblasts, which were formed as a result of satellite cell divisions in the postnatal period [10]. The satellite cell proliferation process taking place in the juvenile growth period is highly important as it enables elongation of muscle fibers. Muscle growth occurring through hypertrophy in the postnatal period is primarily associated with an increase in the amount of nuclear DNA through the constant attachment of satellite cells to the growing muscle fibers. The increased amount of DNA in muscle fiber cells is responsible for the intensification of the protein synthesis process, which is followed by hypertrophy of the maturing muscle fibers [11].

Satellite cells are characterized by expression of the paired box transcription factor 7 (Pax7), required for the survival of satellite cells and prevention of premature differentiation of myogenic progenitor cells [12,13]. It turns out that Pax7 overexpression inhibits myogenesis through inhibition of MyoD expression and induction of myogenin, preventing the differentiation of muscle cells [12,14]. Determination and differentiation pathways are controlled by the MyoD family of myogenic regulatory factors (MRFs), including myogenic factor 5 (MYF5), myoblast determination protein 1 (MYOD), myogenin (MYOG), and myogenic regulatory factor 4 (MRF4), which coordinate with the myocyte enhancer factor-2 family factors to synergistically activate the transcription of genes specific for muscles through recruitment of the chromatin remodeling protein [15].

The aim of the study was to identify differences in transcriptomic profiles of primary muscle cell cultures of cattle breeds of varying performance. Our research hypothesis assumed that the differences in the cattle muscle phenotype are determined already at the transcriptome level of proliferating myogenic cells, which are the source of myoblasts fusing with muscle fibers during their growth and maturation. Better understanding of the factors responsible for the development of muscle tissue, as well as determination of the genes of key importance from the standpoint of the myogenesis process and muscle maturation, will enable determination of those responsible for greater growths of muscle tissue in bulls of beef breeds.

## 2. Results

### 2.1. Course of In Vitro Primary Culture of Muscle Cells

#### 2.1.1. Primary Skeletal Muscle Cell Culture

The microscopic observation of a continuous primary culture of muscle cells isolated from semitendinosus muscle collected from bulls of three cattle breeds, revealed on days 6, 10, and 14 of culture the differences in the rate and progress of cell division between the studied beef breeds (LIM and HER) and the dairy breed (HF). A higher number of proliferating muscle cells was observed in the culture of beef breeds (Figure S1).

#### 2.1.2. Differentiation Culture

On individual days of differentiation, considerably more intense formation of myotubes was observed in beef breeds compared to dairy breed. On the second day of differentiation, myoblasts aligned longitudinally, assumed a fusiform shape, and began to adhere to each other in beef breeds, indicating the start of the cell fusion into myotubes, whereas in the dairy breed, no elongated myoblasts were present. In the case of the culture of LIM and HER breeds, myotubes formed as a result of myoblasts fusion were perceptible already from the fourth day of differentiation, whereas the myotube formation in the HF breed can be only observed on the sixth day of differentiation (Figure S2). Previously published research performed on a similar primary skeletal muscle cell culture derived from the same individuals showed statistically significantly higher numbers of myotubes in beef breeds in comparison to the dairy breed [16].

The presence of a myosin heavy chain (MyHC) was determined in randomly selected cultures of cattle muscle cells, which is a marker of myogenic differentiation. Photographs taken using confocal microscope after six days of muscle cell differentiation (Figure S3) showed clearly formed multinucleated myotubes in individuals of the LIM, HER, and HF breeds, as well as the presence of myosin heavy chains in the myotubes. In addition, a higher number and greater size of myotubes were observed in beef breeds than in the dairy breed (Figure S3).

### 2.2. Transcriptomic and Ontological Analysis

**Figure 1:**
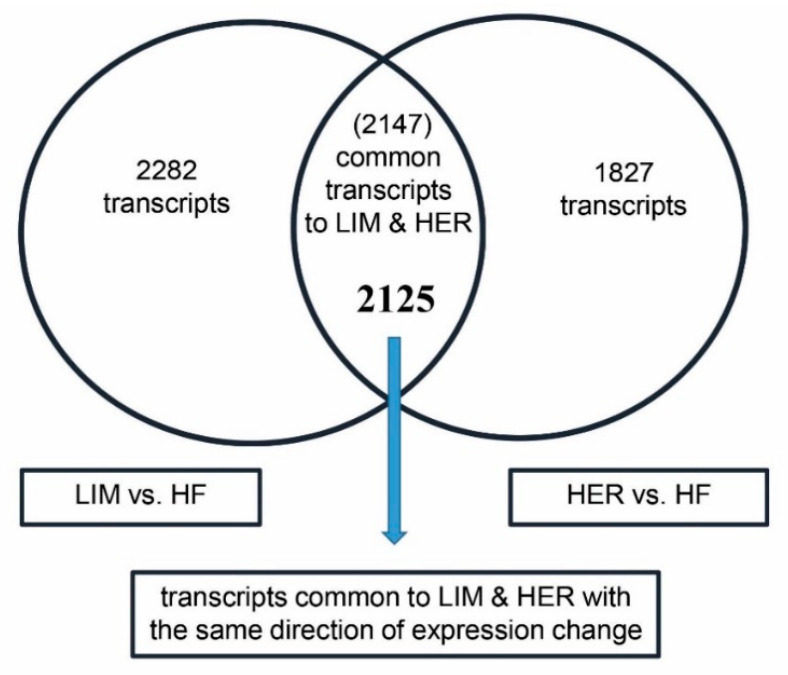
The number of transcripts identified as part of the comparison between primary cultures of semitendinosus muscle cells of beef breeds (Limousin (LIM) and Hereford (HER)) relative to the dairy breed (Holstein-Friesian (HF)), showing statistically significant differences in expression (*p* ≤ 0.05; fold change (FC) ≥ 1.3) (*n* = 4 for each breed).

The transcriptomic analysis allowed identification of 2147 common transcripts for muscle cells of beef breeds relative to the dairy breed, with statistically significant differences in expression (*p* ≤ 0.05; FC ≥ 1.3). A total of 2125 common transcripts, showing expression change in the same direction in both beef breeds, were selected for further analyses (Figure 1). Such a selection criterion will enable identification of the key genes involved in myogenesis regulation in beef cattle, thus enabling determination of the genes conditioning the cattle phenotype with greater muscle mass growth. Of the 2125 identified transcripts, 825 were characterized by greater expression and 1300 by lower expression in the case of LIM and HER bulls, relative to HF bulls. Finally, the subsequent ontological analyses took into account all nonduplicated genes common for beef breeds (1413), with the same direction of expression change, the expression of which was subject to at least a 1.3-fold change in both comparisons.

**Figure 2:**
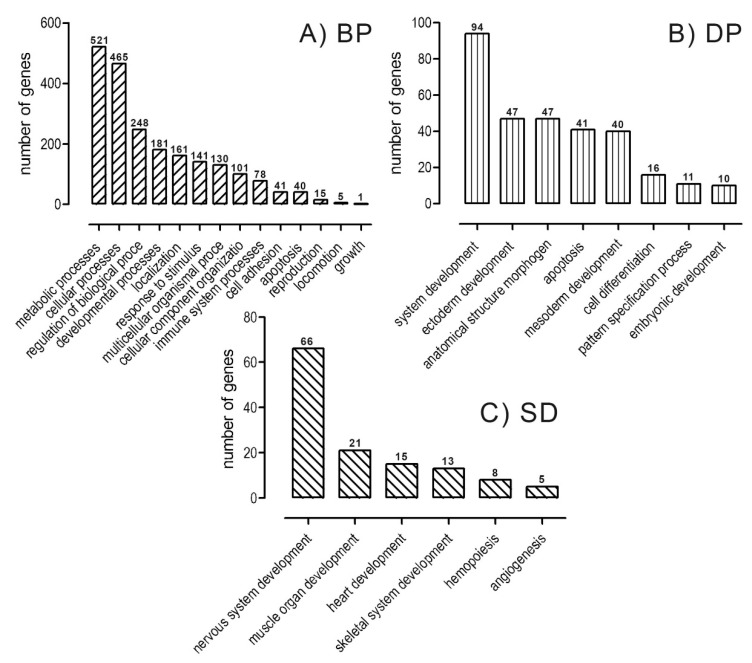
Functional classification of the identified genes that differed statistically significantly (*p* ≤ 0.05) in expression between proliferating muscle cells of 15-month-old bulls from beef breeds (LIM and HER) and HF, a dairy breed, in terms of their involvement (**A**) in biological processes (BP), (**B**) developmental processes (DP) and (**C**) system development (SD). Analysis was performed using the *Panther 7.0* software.

Analysis of the identified common genes (1413) was performed to determine their involvement in biological processes. Ontological analysis demonstrated that the genes which had significant change in their expression are associated with 14 biological processes (Figure 2A), 8 developmental processes (Figure 2B), and 6 system developments (Figure 2C). The process associated with muscle organ development was represented by 21 genes (Table S1).

In addition, an ontological analysis assigned the common genes identified from proliferating muscle cells of beef breed bulls to 601 biological processes (*p* ≤ 0.05; Pathway Studio). Table S2 demonstrates only those biological processes that are associated with the skeletal muscle organ development and additionally includes processes occurring in smooth and cardiac muscles.

For a better and more detailed understanding of the processes, with which the genes common for LIM and HER are associated, an additional, supplementary ontological analysis was performed (Database for Annotation, Visualization, and Integrated Discovery—DAVID) and assigned the selected common genes to 10 biological processes associated with muscle development, enabling a more detailed interpretation of the results (Table S3).

Further analyses took into account the group of genes common for LIM and HER breeds which were assigned by the DAVID to the process associated with muscle organ development. This process was represented by a group of 43 genes presented in Table 1.

**Figure 3:**
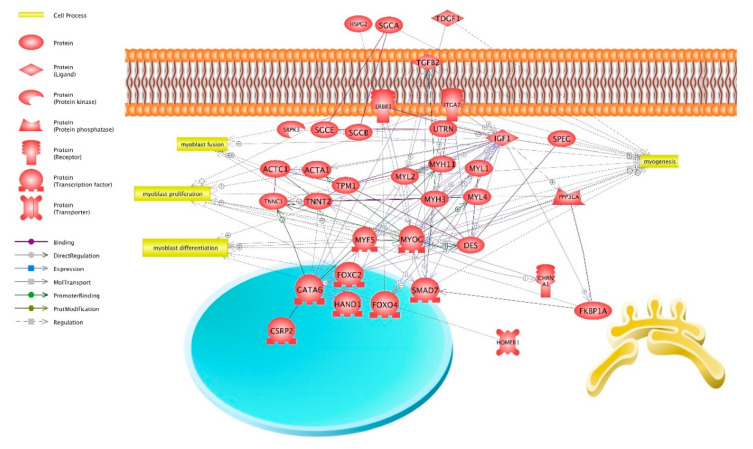
Intergene interaction network of identified common genes for beef breeds and their classification to biological processes associated with muscle organ development (*Pathway Studio*).

A network of relationships of the identified genes common for LIM and HER was created showing intergene interactions and their classification to the following biological processes linked to muscle organ development: myogenesis (17 genes), myoblast proliferation (9), myoblast differentiation (6), and myoblast fusion (5) (Figure 3).

### 2.3. Result Verification with the Use of qPCR Method

**Figure 4:**
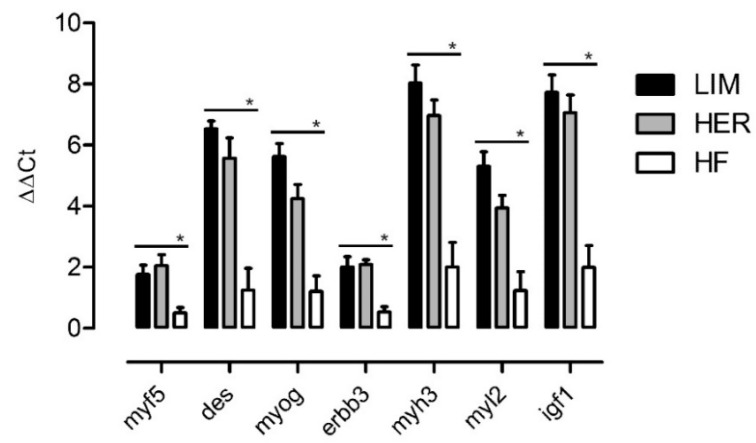
qPCR verification of selected genes associated with muscle organ development. The obtained results were statistically elaborated using one-way analysis of variance and Tukey’s multiple range test. Results are presented as mean ± standard error and are marked with asterisk * for *p* < 0.05; *n* = 4 for each breed; *gapdh*—reference gene; *GraphPad Prism 5* (GraphPad Software Inc., USA).

For the purposes of results verification, seven genes were selected, demonstrating similar changes of expression in both beef breeds as compared with the dairy breed and associated with the process of muscle organ development. Results obtained using the real-time polymerase chain reaction (qPCR) method demonstrated a higher level of expression in all examined genes linked to the development of the muscle organ: *myf5*; desmin (*des*); *myog*; erb-b2 receptor tyrosine kinase 3 (*erbb3*); myosin heavy chain (*myh3*); myosin light chain (*myl2*); and insulin like growth factor 1 (*igf-1*) in the primary culture of cells isolated from the semitendinosus muscle in beef breed bulls (Figure 4). These results confirm those obtained from the microarray analysis.

## 3. Discussion

The present study focused on a group of genes related to muscle organ development. Statistically significant differences observed in their expression between beef breed bulls and dairy breed bulls (Table 1) may point to their significant involvement in the process of growth and development of muscles in beef breeds. We developed a network of relationships of the identified genes common for LIM and HER, assigned by the DAVID to the process associated with muscle organ development (Figure 3): myogenesis (17 genes: *des*; *erbb3*; forkhead box C2 (*foxc2*); GATA binding protein 6 (*gata6*); *igf-1*; integrin alpha 7 (*itga7*); *myf5*; *myl2*; *myh3*; *myog*; sarcoglycan alpha (*sgca*); SMAD family member 7 (*smad7*); striated muscle enriched protein kinase (*speg*); SRSF protein kinase 3 (*srpk3*); teratocarcinoma-derived growth factor 1 (*tdgf1*); transforming growth factor beta 2 (*tgf-β2*); utrophin (*utrn*)); myoblasts proliferation (9 genes: *des*; *foxc2*; FKBP prolyl isomerase 1A (*fkbp1a*); *igf-1*; *itga7*; *myf5*; *myog*; protein phosphatase 3 catalytic subunit alpha (*ppp3ca*); *tgf-β2*); myoblasts differentiation (6 genes: *des*; *foxc2*; homer scaffold protein 1 (*homer1*); *igf-1*; *myf5*; *myog*); and myoblasts fusion (5 genes: *des*, *erbb3*, *igf-1*, *myf5*, *myog*) (Figure 3).

The first genes identified and validated by qPCR are *myf5* and *myog* (Figure 4, Table 1). Both were assigned to processes associated with myogenesis, myoblast proliferation, myoblast differentiation, and myoblast fusion (Figure 3). It is well-known that *myf5* and myogenin belong to MRFs and play a significant role in the development and growth of skeletal muscles. This regulation consists of activation of quiescent satellite cells, their proliferation, differentiation, and fusion into multinucleated myotubes, maturing into functional muscle fibers [7,8,10].

*Myf5* is a muscle-specific factor, which undergoes expression at the earliest stage during the development of skeletal muscles, and its expression is necessary to direct myogenic cells [17,18]. Activated satellite cells exhibit *myf5* expression, as it is the first factor that promotes their proliferation [19]. In numerous studies, a higher level of *myf5* expression in proliferating muscle cells was observed as well as a decrease in the number of cells as a result of *myf5* downregulation [20,21,22]. In the study conducted by Coles et al. [23], a higher level of *MYF5* expression and higher proliferation potential of myoblasts originating from the muscle was observed in Angus and HER breeds than in the Wagyu breed and was positively correlated with higher muscle mass in these breeds. Consequently, the higher *myf5* expression level in proliferating cells from beef bulls demonstrated herein (Figure 4, Table 1) may further contribute to the higher muscle mass growths in individuals of beef breeds, which are characterized by a significantly higher share of muscle in carcasses, and higher dressing percentage and content of valuable cuts than the dairy breed [24,25].

Myogenin plays a key role in the development of skeletal muscles during the process of myoblast differentiation. As a result of induction of differentiation, myogenin translocates from the cytoplasm to the nucleus, which allows cells to begin this process [26]. Myogenin expression, despite being lower in proliferating than in differentiating myoblasts, begins before the cells leave the cellular cycle and enter the postmitotic stage, determining fusion with neighboring cells [20,27,28,29,30]. The ultimate differentiation of myogenic progenitor cells is characterized by early MYOG expression, followed by myofibrillar protein expression, such as the myosin heavy chain, right before fusion with the forming myotubes [31,32]. Myogenin expression was also demonstrated in proliferating and differentiating bovine satellite cells [33,34]. It is likely that at least some of the tested cells isolated from semitendinosus muscle in LIM and HER breeds of cattle had already commenced differentiation which determines fusion into multinucleated myotubes, indicating increased expression of myogenin and other genes described in the following part of the Discussion, including *myl2*, *myh3*, actin alpha 1 skeletal muscle (*acta1*), actin alpha cardiac muscle 1 (*actc1*), tropomyosin 1 (*tpm1*), troponin T2, cardiac type (*tnnt2*), and troponin C type 1, slow (*tnnc1*). These genes play a key role in the differentiation and fusion of muscle cells, and also have a positive impact on the process of myogenesis of skeletal muscles [35,36,37,38].

Higher *myog* expression in beef breeds may also stem from the elevated *igf-1* expression, which was upregulated in beef breeds (Figure 4, Table 1). IGF-1 stimulates MRFs such as MyoD, myogenin, and MYH3 [39,40,41,42]. According to functional analysis, *igf-1* was assigned to the following biological processes: myogenesis, myoblast proliferation, myoblast differentiation, and myoblast fusion (Figure 3). In addition, IGF-1 is considered to be a factor involved in muscle tissue hypertrophy and regeneration processes [41,43]. An elevated IGF-1 level stimulates the synthesis of skeletal muscle proteins, primarily myofibrils such as actins, troponins, tropomyosins incorporated into thin myofilaments, and myosin included in thick myofilaments. These proteins comprise approximately 80% of the sarcoplasm of muscle fibers and participate in the construction of sarcomere. Elevated synthesis of structural proteins contributes to better growth of muscle tissue [44,45]. It is worth noting that various expression levels of IGF1 may result from DNA mutations and differences in single nucleotide polymorphisms (SNPs) prevalence between the examined breeds. Previous studies have shown the effect of the SNP of the IGF-1 gene on the growth rate and meat production traits in beef cattle such as: Limousin and Hereford [46], Angus [47,48], Montbeliarde [49], and Hanwoo [50]. It is possible that the higher *igf-1* expression level in LIM and HER bulls compared with HF further contributes to achieving higher muscle mass growth in typical beef breeds.

Results of the transcriptomic analysis performed as part of the present study demonstrated that muscle cells of beef cattle are characterized by a higher expression level of genes associated with myosin heavy chains (*myh3* and *myh11*) as well as genes associated with myosin light chains (*myl1*, *myl2*, *myl4*) (Figure 4, Table 1). These genes were classified to the myogenesis process (Figure 3). Myosin light chains (MYL1, MYL2) constitute myosin complex components and can act as a molecular motor providing energy to muscle contraction [51]. An elevated expression level of *myh3* and *myl2* genes was identified during the differentiation of cattle [33,52], mouse [53], and human satellite cells [54]. On the contrary, lowered expression of *MYH3* and *MYL2* was shown in muscle cells deprived of myogenin [33]. Myosin heavy chains 3 (MYH3) undergoes expression in skeletal muscle cells, promotes cell fusion into myotubes [35,55], and its expression is positively correlated with higher growth of muscle mass in cattle [56,57]. Similar relationships were observed in the present study, with a higher *myh3* expression level in beef bulls (Figure 4, Table 1). Additionally, a reduced *myh3* level was observed in myoblasts with lowered IGF-1 expression, however, the administration of IGF-1 to the medium resulted in elevated *Myh3* expression [58]. It is possible that elevated myogenin and *igf-1* expression necessary for the normal course of myogenesis, also identified in LIM and HER bulls, are responsible for increased *myh3* and *myl2* expression and may influence muscle development in bulls from beef breeds.

Other genes that may constitute significant regulators of growth and development of skeletal muscles are *acta1* and *actc1*, *tpm1*, *tnnc1*, and *tnnt2* (Figure 3, Table 1). The elevated expression level of the aforementioned muscle-specific genes in myoblasts differentiating into multinucleated myotubes was confirmed [59,60,61,62] and may be responsible for an elevated level of proteins forming a contractile apparatus, thus contributing to a more intensive hypertrophy of muscle tissue observed in beef breeds at a later stage of skeletal muscle development. The expression of genes encoding proteins of the contractile apparatus may be stimulated by the aforementioned myogenin and IGF-1 [63,64]. It is a known fact that in the early stages of myoblast differentiation, the MyoD+/MYOG+ myocytes begin to accumulate muscle-specific ACTA1 and MYH3. Subsequently, myocytes may fuse, forming α-actinin+/MYOG+ multinucleated myotubes, and finally, muscle fibers [65].

Another gene identified via microarray analysis, the expression of which was higher in cells, originating from beef breeds is transforming growth factor, beta 2 (*tgf-β2*) (Table 1). *Tgf-β2* was classified to biological processes associated with myogenesis and myoblast proliferation (Figure 3). *Tgf-β2* is a factor belonging to the TGF-β superfamily, which regulates skeletal muscle growth, stimulating the proliferation of skeletal muscle satellite cells [66]. Rudolf et al. [67] demonstrated that exogenous TGF-β2 stimulation restores muscle regeneration potential by disturbing the level of β-catenin in satellite cells. Szcześniak et al. [68] revealed that the expression of the *tgf-β2* gene was downregulated in equine satellite cells (ESC) treated with β-hydroxy-β-methyl butyrate (HMB). However, this study was conducted on differentiating cells.

Desmin, which showed higher expression in muscle cells of beef breed individuals, is another important gene involved in the process of muscle organ development (Figure 4, Table 1). Functional analysis demonstrated its association with the myogenesis, proliferation, differentiation, and fusion of myoblasts (Figure 3). Desmin is the key protein of the muscle cell cytoskeleton, located on thin (actin) myofilaments, and responsible for the normal function of muscle cells, including integrity of the internal environment and regulation of the location and function of the contractile apparatus, cell nucleus, and mitochondria [69]. Desmin is a muscle-specific protein expressed at the beginning and the end of the myogenic program and accumulated during in vitro myogenesis and is one of the key markers of muscle differentiation [70,71]. During myogenesis, desmin is subject to expression in undifferentiated muscle cells, myotubes, and muscle fibers, and its expression precedes that of other proteins of the contractile apparatus, such as actin, myosin, troponin, and tropomyosin and also precedes the expression of muscle-specific genes, such as *myod*, *myf5*, *myog*, and *mrf4* [72,73]. Therefore, such an early appearance of desmin mRNA confirms its key role in the development and function of muscle cells. Moreover, Yu et al. [19] demonstrated that IGF-1 administration considerably increased the expression of myogenic factors, including desmin. Higher desmin expression in LIM and HER bulls may depend on the higher *igf-1* expression in beef breeds (Table 1).

The last identified gene that was classified to myogenesis processes and myoblast fusion, the expression of which was higher in muscle cells in beef breeds, is *erbb3* (Figure 3 and Figure 4, Table 1). *Erbb3* encodes the tyrosine surface receptor for neuregulin (NRG) also expressed in cultured myotubes. NRG participates in several processes associated with the development of skeletal muscles, such as myogenesis, muscle fiber survival, and muscle spindle development [74,75]. Muscle cells commencing the differentiation process secrete neuregulin that is necessary for the induction of the early stage of differentiation [76,77]. As a myogenic factor, NRG promotes myogenin expression, thus stimulating myoblasts for withdrawal from the cellular cycle and beginning differentiation, inducing fusion to multinucleated myotubes and formation of the muscle spindle through an increase in MyHC expression [78,79,80]. It has been revealed that NRG increases protein synthesis, contributing to the regulation of skeletal muscle mass [79,81]. On the contrary, Sadkowski et al. [24] revealed a decreased level of *erbb3* expression in the tissue of semitendinosus muscle in LIM and HER bull breeds as in the HF breed. However, it should be emphasized that the study was carried out on a fully mature muscle tissue, with an established number of muscle fibers. Thus, it seems that the higher *erbb3* expression observed in muscle cells from both beef breeds (Figure 4, Table 1) is associated with proliferation and may be highly important for the regulation of this process.

**Figure 5:**
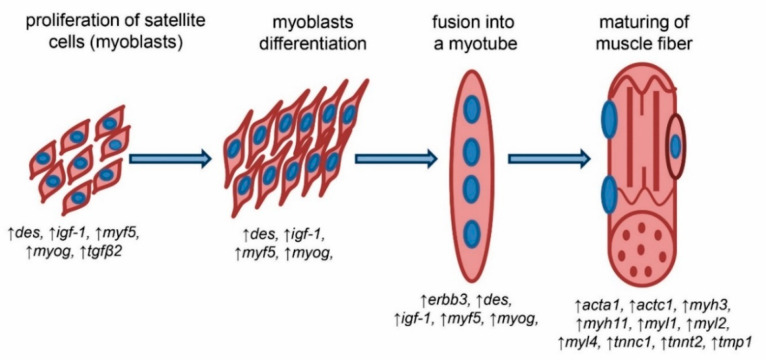
Involvement of the identified genes in processes associated with the development and maturation of muscle fibers.

In conclusion, a higher expression level of genes identified in the cell cultures isolated from semitendinosus muscle of LIM and HER breeds may indicate their significant role in the processes of the formation and maturation of skeletal muscle in beef cattle. Hence, it is plausible that the increased differentiation stimulation by key genes associated with the general myogenesis process such as *myf5*, *myog*, *des*, *igf-1*, *erbb3*, and *tgfb2*, and elevated contractile apparatus proteins levels such as *acta1*, *actc1*, *myh3*, *myl2*, *tpm1*, *tnnt2*, and *tnnc1* responsible for building a significant portion of the muscle fiber mass, is the mechanism that allows the greater growth and development rate of muscle tissue in cattle classified as beef breeds. It needs to be highlighted that myogenin and IGF-1 could be the main factors orchestrating the aforementioned genes by the stimulation of differentiation progression and protein synthesis necessary for higher muscle mass growth. As a summary, the involvement of the identified genes in the distinct processes associated with the development and maturation of muscle fibers with a fully developed contractile apparatus is presented in Figure 5.

This study extends the current knowledge about genes’ involvement in the myogenesis process and describes their effect on the skeletal muscle development in the studied cattle breeds. The results of this study can be useful in selecting cattle towards highly efficient beef production which may have economic significance.

## 4. Materials and Methods 

### 4.1. Ethics Statement

This study complies with national and institutional guidelines of the use of animals in research according to the Polish Legal Act of January 21, 2005. Since sample collection was performed during routine slaughter and no additional procedures that were harmful or painful for the animals were applied, this study did not require a formal ethics approval.

### 4.2. Animals and Cell Samples

Transcriptome analyses were conducted on primary cultures of skeletal muscle cell isolated from semitendinosus muscle of 15-month-old bulls. The experimental group was composed of four bulls of each breed of varying performance (HER and LIM beef bulls; HF dairy bulls—a reference). The bulls were housed, fed, and slaughtered at the age of 15 months as described earlier [16,82].

Samples of semitendinosus muscle were immediately taken after the slaughter of the animals in an abattoir and washed four times in phosphate-buffered saline (PBS, Sigma-Aldrich, Saint Louis, MO, USA) with an antibiotic at decreasing concentration (40,000 and 20,000 units Penicillinum crystallisatum /100 mL PBS) (Penicillinum crystallisatum TZF; Polfa Tarchomin, Warsaw, Poland). Next, skeletal muscle samples were cleaned from connective and adipose tissue and cut into small pieces using sterile surgical instruments. Fragmented samples were suspended in sterile fetal bovine serum (FBS) with 10% dimethyl sulfoxide (DMSO) (Sigma-Aldrich, Saint Louis, MO, USA). The prepared samples were gradually cooled to −80 °C and then stored in liquid nitrogen until satellite cell isolation.

### 4.3. Skeletal Muscle Cells Isolation, Proliferation, and Differentiation

To isolate muscle cells, samples of semitendinosus muscle were thawed immediately in a water bath and subsequently washed in PBS with Penicillinum crystallisatum. After PBS aspiration, the incubation medium (Dulbecco’s modified Eagle medium (DMEM) (Gibco, Life Technologies, Carlsbad, CA, USA), Pronase from *Streptomyces griseus* (Sigma-Aldrich, Saint Louis, MO, USA) at 0.5 mg/mL, 10% FBS, Penicillinum crystallisatum) was added. Samples were incubated for 1.5 h at 37 °C with constant mixing. Afterwards, the suspension with isolated muscle cells was sieved through a 70-µm nylon filter to separate tissue debris. The filtrate-containing cells were centrifuged three times and resuspended in proliferation medium (10%FBS/DMEM/1% penicillin–streptomycin and 0.5% amphotericin B (Gibco, Life Technologies, Carlsbad, CA, USA)) and transferred to Primaria tissue culture flasks (Becton Dickinson, Franklin Lakes, NJ, USA). The 1-h preplating applied four times finally allowed for achieving 60–70% myoblast purity [16]. Next, 100,000 cells were transferred to culture flasks. Isolated muscle cells of beef and dairy breeds were cultured in proliferation medium until 80% of confluence, which was changed every 48 h. Then, the cells were trypsinized and centrifuged, the supernatant was discarded, and the cell pellet was frozen for further analysis.

### 4.4. Immunocytochemical Analysis

A part of the isolated cells was cultured to obtain 90% of confluence. Then, the proliferation medium was replaced with a differentiation medium (2% horse serum-HS (Gibco, Life Technologies, Carlsbad, CA, USA)/DMEM/1% penicillin–streptomycin and 0.5% amphotericin B) in the next 6 days in order to validate the type of isolated cells, using immunofluorescence, and carry out morphological analysis of the culture. The differentiation medium was replaced every 48 h. After the sixth day, the cells were fixed with 0.25% paraformaldehyde (Sigma-Aldrich, Saint Louis, MO, USA) for 30 min, washed with PBS, and incubated in ice-cold 70% methanol. Then, cells were incubated with rabbit primary anti-MyHC antibody (Santa Cruz Biotechnology, Dallas, TX, USA) diluted 1:100 with PBS for 1 h. In addition, anti-rabbit secondary antibody conjugated with Alexa Fluor 488 fluorescent dye (Sigma-Aldrich, Saint Louis, MO, USA) suspended in PBS (1:500 dilution) was added and incubated for 1 h. To stain the nuclei, the cells were incubated for 30 min in a solution of 7-aminoactinomycin D (7AAD; Sigma-Aldrich, Saint Louis, MO, USA) (5 mg/mL). The cells were observed using the FV-500 confocal laser scanning microscope (Olympus Optical Co., Hamburg, Germany).

### 4.5. RNA Isolation and Validation

Total RNA was isolated from muscle cells using a Total RNA kit (A&A Biotechnology, Gdynia, Poland) according to the manufacturer’s instructions. Samples were validated using a Nanodrop spectrophotometer (Nanodrop Technologies, Wilmington, DE, USA) and Bioanalyzer 2100 (Agilent Technologies, Santa Clara, CA, USA). The eluted RNA was stored at −80 °C until further analysis. Only samples with RNA Integrity Number (RIN) > 9 were included in further analysis.

### 4.6. Gene Expression Analysis

Gene expression was evaluated using Bovine (V2) Gene Expression Microarray 4×44K oligonucleotide slides (Agilent Technologies, Santa Clara, CA, USA) and Two-Color Microarray-Based Gene Expression Analysis kit (Agilent Technologies, Santa Clara, CA, USA). Probe labeling, hybridization, signal detection, and data extraction were performed as described in a previous paper [83]. Differentially expressed genes were identified by the Gene Spring 13.0 software (Agilent Technologies, Santa Clara, CA, USA) using t-test with *p* ≤ 0.05 and fold change (FC) ≥ 1.3 as the criteria of significance. The data obtained in the microarray experiment were deposited in the National Center for Biotechnology Information (NCBI) Gene Expression Omnibus database (GEO) and numbered GSE151274. Sequences that were not assigned a gene name by the microarray manufacturer were compared with the NCBI Blastn Nucleotide base. Sequences not showing 100% complementarity with eukaryotic mRNA were excluded from further analyses.

### 4.7. qPCR Validation

To validate the microarray results, selected genes were examined using the qPCR technique. The mRNA sequences of genes were obtained from the NCBI Nucleotide database. Primer sequences were designed using the Primer3 (http://frodo.wi.mit.edu/primer3/) and Primer-Blast software (http://www.ncbi.nlm.nih.gov/tools/primer-blast/) and then checked using Oligo Calculator (http://www.basic.northwestern.edu/biotools/oligocalc.html) as described previously [84]. Primer sequences, annealing temperatures, and product length measurements are listed in Table S4. Glyceraldehyde-3-phosphate dehydrogenase (*gapdh*) was used for normalization as the reference gene [85]. qPCR was performed according to an earlier described methodology [86]. Results were calculated using the Livak method [87] and presented in ΔΔ*C*T as the ratio of verified target gene expression to the expression of the reference gene (*gapdh*) that was calculated as the arithmetic mean.

### 4.8. Statistical Analysis

Statistical analysis of microarray data was performed using the Gene Spring 13.0 software (Agilent Technologies, Santa Clara, CA, USA). The mRNA qPCR results were analyzed using Prism 5.0 (GraphPad Software, La Jolla, CA, USA). Statistical significance was checked by one-way analysis of variance (ANOVA) with Tukey’s post hoc testing considering *p*-value ≤ 0.05 as significant. The data are shown as mean ± standard error.

### 4.9. Functional Analysis

The results were subjected to ontological analysis using the following online available databases: Panther Classification System 7.0 (http://www.pantherdb.org) using a statistical overrepresentation test with Bonferroni correction; Database for Annotation, Visualization, and Integrated Discovery (DAVID v6 database 7; https://david.ncifcrf.gov/; with the Benjamini and Hochberg test correction (false discovery rate—FDR)). Pathway Studio Web (Elsevier, Amsterdam, Netherlands; https://mammalcedfx.pathwaystudio.com/) was used for the intergene interaction network design. In addition, during the development of data from microarrays, numerous internet databases, which are grouped in NCBI, such as GenBank, OMIM, PubMed, and iHOP, were used.

## Figures and Tables

**Table 1:** List of identified common genes associated with muscle organ development (Database for Annotation, Visualization, and Integrated Discovery—*DAVID*).

No.	Gene symbol	Gene name (GenBank Accession Number)	LIM vs. HF FC	HER vs. HF FC
1	*acta1*	Bos taurus actin, alpha 1, skeletal muscle (ACTA1), mRNA [NM_174225]	26.71	25.31
2	*unc45b*	unc-45 homolog B (C. elegans) [Source:HGNC Symbol;Acc:14304] [ENSBTAT00000003766]	22.15	10.91
3	*myh3*	Bos taurus myosin, heavy chain 3, skeletal muscle, embryonic (MYH3), mRNA [NM_001101835]	19.69	16.58
4	*myl2*	Bos taurus myosin, light chain 2, regulatory, cardiac, slow (MYL2), mRNA [NM_001035025]	15.10	8.60
5	*col11a1*	Bos taurus collagen, type XI, alpha 1 (COL11A1), mRNA [NM_001166509]	14.60	8.95
6	*actc1*	Bos taurus actin, alpha, cardiac muscle 1 (ACTC1), mRNA [NM_001034585]	12.39	8.16
7	*eln*	elastin [Source:HGNC Symbol;Acc:3327] [ENSBTAT00000057593]	12.10	8.66
8	*tnnt2*	Bos taurus troponin T type 2 (cardiac) (TNNT2), mRNA [NM_174771]	11.90	12.98
9	*myl1*	Bos taurus myosin, light chain 1, alkali; skeletal, fast (MYL1), mRNA [NM_001079578]	11.56	8.76
10	*igf1*	PREDICTED: Bos taurus insulin like growth factor 1 (IGF1), transcript variant X8, mRNA [XM_005206500]	11.10	13.75
11	*ttn*	titin [Source:HGNC Symbol;Acc:12403] [ENSBTAT00000061449]	9.10	4.27
12	*tnnc1*	Bos taurus troponin C type 1 (slow) (TNNC1), mRNA [NM_001034351]	8.07	5.69
13	*des*	Bos taurus desmin (DES), mRNA [NM_001081575]	7.51	5.54
14	*hspg2*	PREDICTED: Bos taurus heparan sulfate proteoglycan 2 (HSPG2), mRNA [XM_582024]	7.07	4.37
15	*myf5*	Bos taurus myogenic factor 5 (MYF5), mRNA [NM_174116]	5.32	2.61
16	*sgca*	PREDICTED: Bos taurus sarcoglycan alpha (SGCA), transcript variant X1, mRNA [XM_005220623]	4.90	4.57
17	*tpm1*	Bos taurus tropomyosin 1 (alpha) (TPM1), mRNA [NM_001013590]	4.87	4.38
18	*myl4*	Bos taurus myosin, light chain 4, alkali; atrial, embryonic (MYL4), mRNA [NM_001075149]	4.26	3.43
19	*myog*	Bos taurus myogenin (myogenic factor 4) (MYOG), mRNA [NM_001111325]	4.11	4.19
20	*tagln3*	Bos taurus transgelin 3 (TAGLN3), mRNA [NM_001034499]	3.38	2.97
21	*speg*	Bos taurus SPEG complex locus, mRNA (cDNA clone IMAGE:8085922), partial cds, [BC113258]	3.36	3.57
22	*itga7*	Bos taurus integrin, alpha 7 (ITGA7), mRNA [NM_001191305]	3.10	2.64
23	*erbb3*	Bos taurus v-erb-b2 erythroblastic leukemia viral oncogene homolog 3 (avian) (ERBB3), mRNA [NM_001103105]	2.91	1.57
24	*myl6b*	Bos taurus myosin, light chain 6B, alkali, smooth muscle and non-muscle (MYL6B), mRNA [NM_001075713]	2.91	2.33
25	*homer1*	PREDICTED: Bos taurus homer scaffolding protein 1 (HOMER1), transcript variant X2, mRNA [XM_015473042]	2.83	3.15
26	*sgce*	Bos taurus sarcoglycan, epsilon (SGCE), mRNA [NM_001075145]	2.59	2.11
27	*tgfb2*	Bos taurus transforming growth factor, beta 2 (TGFB2), mRNA [NM_001113252]	2.57	2.39
28	*chrna1*	Bos taurus cholinergic receptor, nicotinic, alpha 1 (muscle) (CHRNA1), mRNA [NM_176664]	2.46	2.82
29	*foxo4*	Bos taurus forkhead box O4 (FOXO4), mRNA [NM_001101277]	2.44	2.28
30	*myh11*	Bos taurus myosin, heavy chain 11, smooth muscle (MYH11), mRNA [NM_001102127]	2.24	3.88
31	*myl6*	Bos taurus myosin, light chain 6, alkali, smooth muscle and non-muscle (MYL6), mRNA [NM_175780]	2.18	1.62
32	*sgcb*	Bos taurus sarcoglycan, beta (43kDa dystrophin-associated glycoprotein) (SGCB), mRNA [NM_001102188]	2.13	1.83
33	*fkbp1a*	Bos taurus FK506 binding protein 1A, 12kDa, mRNA (cDNA clone IMAGE:7951983), partial cds. [BC102338]	2.05	1.80
34	*utrn*	Bos taurus utrophin (UTRN), mRNA [NM_001278561]	1.95	2.26
35	*hand1*	Bos taurus heart and neural crest derivatives expressed 1 (HAND1), mRNA [NM_001075761]	−2.84	−1.80
36	*csrp2*	Bos taurus cysteine and glycine-rich protein 2 (CSRP2), mRNA [NM_001038183]	−2.59	−2.34
37	*gata6*	GATA binding protein 6 [Source:HGNC Symbol;Acc:4174] [ENSBTAT00000007537]	−2.51	−1.75
38	*srpk3*	Bos taurus SRSF protein kinase 3 (SRPK3), mRNA [NM_001083390]	−2.27	−1.69
39	*smad7*	PREDICTED: Bos taurus SMAD family member 7 (SMAD7), transcript variant X1, mRNA [XM_005224231]	−2.27	−2.11
40	*ppp3ca*	Bos taurus protein phosphatase 3, catalytic subunit, alpha isozyme (PPP3CA), mRNA [NM_174787]	−1.73	−1.79
41	*tdgf1*	Bos taurus teratocarcinoma-derived growth factor 1 (TDGF1), mRNA [NM_001080358]	−1.72	−1.58
42	*foxc2*	Bos taurus forkhead box C2 (MFH-1, mesenchyme forkhead 1) (FOXC2), mRNA [NM_001193072]	−1.61	−1.49
43	*nf1*	Bos taurus neurofibromin 1 (NF1), mRNA, [Source:RefSeq mRNA;Acc:NM_001122728] [ENSBTAT00000015699]	−1.33	−1.49

FC ≥ 1.3; *p* ≤ 0.05; *n* = 4 for each breed. FC, fold change; LIM, Limousin; HER, Hereford; HF, Holstein-Friesian.

## Supplementary Materials

Supplementary materials can be found at https://www.mdpi.com/1422-0067/21/13/4794/s1.

## Abbreviations

7AAD	7-aminoactinomycin D
DAVID	Database for Annotation, Visualization, and Integrated Discovery
DMEM	Dulbecco’s Modified Eagle Medium
DMSO	Dimethyl sulfoxide
FBS	Fetal bovine serum
FC	Fold change
GAPDH	Glyceraldehyde-3-phosphate dehydrogenase
HER	Hereford
HF	Holstein-Friesian
HS	Horse serum
LIM	Limousin
NCBI GEO	National Center for Biotechnology Information Gene Expression Omnibus database
PBS	Phosphate-buffered saline
qPCR	Real-time polymerase chain reaction
RIN	RNA Integrity Number
