# Supplementary material for: Transcriptomic Profile of Primary Culture of Skeletal Muscle Cells Isolated from Semitendinosus Muscle of Beef and Dairy Bulls
Source: Int J Mol Sci. 2020 Jul 7;21(13):4794. doi: 10.3390/ijms21134794 (PMC7369917; doi:10.3390/ijms21134794)
Supplement: Supplementary file 1 [file ijms-21-04794-s001.zip › Figure S1.pdf]

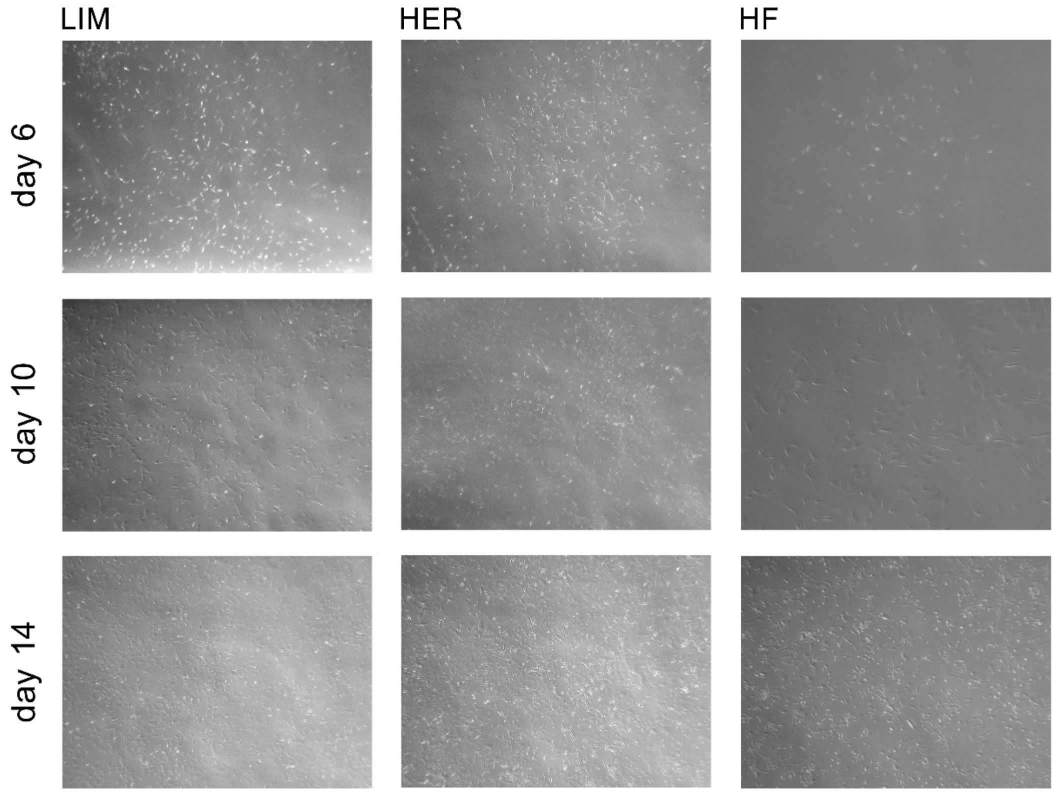

Figure S1. Photographs showing continuous culture (6, 10, and 14 days of proliferation) of muscle cells isolated from semitendinosus muscle of individuals of LIM, HER, and HF breed. The photographs were taken using a phase-contrast microscope, magnification 10×.
