# Supplementary material for: Transcriptomic Profile of Primary Culture of Skeletal Muscle Cells Isolated from Semitendinosus Muscle of Beef and Dairy Bulls
Source: Int J Mol Sci. 2020 Jul 7;21(13):4794. doi: 10.3390/ijms21134794 (PMC7369917; doi:10.3390/ijms21134794)
Supplement: Supplementary file 1 [file ijms-21-04794-s001.zip › Figure S2.pdf]

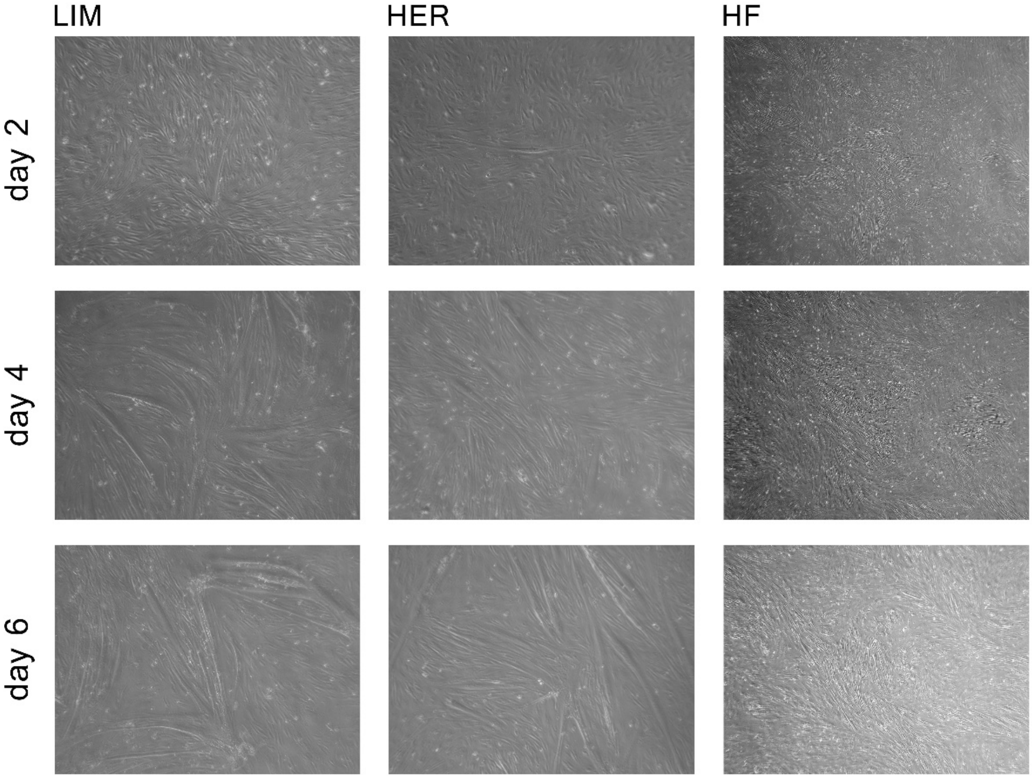


**Figure S2.** Photographs showing individual days of differentiation (2, 4, and 6 days) of muscle cells isolated from the semitendinosus muscle of LIM, HER, and HF bulls. The photographs were taken using a phase-contrast microscope, magnification 10×.
