# Supplementary material for: Transcriptomic Profile of Primary Culture of Skeletal Muscle Cells Isolated from Semitendinosus Muscle of Beef and Dairy Bulls
Source: Int J Mol Sci. 2020 Jul 7;21(13):4794. doi: 10.3390/ijms21134794 (PMC7369917; doi:10.3390/ijms21134794)
Supplement: Supplementary file 1 [file ijms-21-04794-s001.zip › Figure S3.pdf]

LIM HER HF

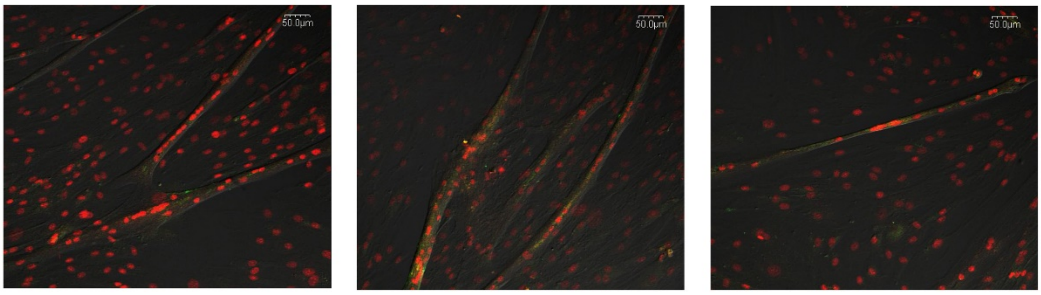

Figure S3. Photographs taken using confocal microscope presenting expression of MyHC (Alexa Fluor488—green fluorescence) with concomitant staining of 7AAD cell nuclei (red fluorescence) performed on the sixth day of differentiation of muscle cells originating from LIM, HER, and HF bulls.
