# Supplementary material for: Transcriptomic Profile of Primary Culture of Skeletal Muscle Cells Isolated from Semitendinosus Muscle of Beef and Dairy Bulls
Source: Int J Mol Sci. 2020 Jul 7;21(13):4794. doi: 10.3390/ijms21134794 (PMC7369917; doi:10.3390/ijms21134794)
Supplement: Supplementary file 1 [file ijms-21-04794-s001.zip › Table S1.pdf]

Table S1. List of identified common genes associated with muscle organ development (*Panther*).

No.	Gene symbol	Gene name (GenBank Accession Number)	LIM vs. HF	HER vs. HF
			FC	FC
1	*cdh13*	Bos taurus cadherin 13, H-cadherin (heart) (CDH13), mRNA [NM_001035277]	22,47	10,56
2	*myl2*	Bos taurus myosin, light chain 2, regulatory, cardiac, slow (MYL2), mRNA [NM_001035025]	15,10	8,60
3	*cdh15*	Bos taurus cadherin 15, type 1, M-cadherin (myotubule) (CDH15), mRNA [NM_001102066]	5,90	4,56
4	*myf5*	Bos taurus myogenic factor 5 (MYF5), mRNA [NM_174116]	5,32	2,61
5	*tpm1*	Bos taurus tropomyosin 1 (alpha) (TPM1), mRNA [NM_001013590]	4,87	4,38
6	*cdh1*	Bos taurus cadherin 1, type 1, E-cadherin (epithelial) (CDH1), mRNA. [Source:RefSeq mRNA;Acc:NM_001002763] [ENSBTAT00000021272]	4,32	3,91
7	*myog*	Bos taurus myogenin (myogenic factor 4) (MYOG), mRNA [NM_001111325]	4,11	4,19
8	*myl9*	Bos taurus myosin, light chain 9, regulatory (MYL9), mRNA [NM_001075234]	2,09	2,18
9	*palld*	Bos taurus palladin, cytoskeletal associated protein, mRNA (cDNA clone IMAGE:8120568), partial cds. [BC104593]	1,98	1,73
10	*pitx3*	Bos taurus paired-like homeodomain 3 (PITX3), mRNA [NM_001192305]	-5,03	-2,01
11	*phox2a*	Bos taurus paired-like homeobox 2a (PHOX2A), mRNA [NM_001192157]	-4,30	-1,84
12	*pcdh1*	Bos taurus protocadherin 1 (PCDH1), mRNA [NM_001083655]	-3,53	-1,91
13	*nkx2-8*	Bos taurus NK2 homeobox 8 (NKX2-8), mRNA [NM_001192163]	-3,49	-1,73
14	*csrp2*	Bos taurus cysteine and glycine-rich protein 2 (CSRP2), mRNA [NM_001038183]	-2,59	-2,34
15	*cdh26*	PREDICTED: Bos taurus cadherin 26 (CDH26), mRNA [XM_869285]	-2,19	-2,08
16	*hmx3*	H6 family homeobox 3 [Source:HGNC Symbol;Acc:5019] [ENSBTAT00000045429]	-1,90	-1,55
17	*zfhx3*	zinc finger homeobox 3 [Source:HGNC Symbol;Acc:777] [ENSBTAT00000019489]	-1,88	-1,92
18	*nkx3-2*	Bos taurus NK3 homeobox 2 (NKX3-2), mRNA [NM_001193133]	-1,85	-1,49
19	*pax1*	paired box 1 [Source:HGNC Symbol;Acc:8615] [ENSBTAT00000008861]	-1,67	-1,45
20	*cdh23*	Bos taurus cadherin-related 23 (CDH23), mRNA [NM_001191206]	-1,58	-2,40
21	*cdhr5*	Bos taurus cadherin-related family member 5 (CDHR5), mRNA [NM_001103326]	-1,46	-1,38

[1] FC ≥ 1.3; $p \leq 0.05$; n = 4 for each breed. FC-fold change; LIM-Limousin; HER-Hereford; HF-Holstein-Friesian.
