# Supplementary material for: Transcriptomic Profile of Primary Culture of Skeletal Muscle Cells Isolated from Semitendinosus Muscle of Beef and Dairy Bulls
Source: Int J Mol Sci. 2020 Jul 7;21(13):4794. doi: 10.3390/ijms21134794 (PMC7369917; doi:10.3390/ijms21134794)
Supplement: Supplementary file 1 [file ijms-21-04794-s001.zip › Table S2.pdf]

Table S2. Classification showing the involvement of genes common for LIM and HER in selected biological processes associated with muscle development (*Pathway Studio*).

Process	Number of genes	Gene symbol	p-value
muscle filament sliding	13	*des; myl6b; tnnt2; myl4; myl2; myl1; actc1; myh3; tnnc2; tpm1; tnnc1; acta1; myl6*	2,04E-08
ventricular cardiac muscle tissue morphogenesis	10	*tnnt2; myl2; hand1; col11a1; smad7; foxc2; foxs1; fkbp1a; tpm1; tnnc1*	1,76E-06
muscle contraction	20	*chrng; des; myl6b; utrn; tnnt2; chrne; myl1; chrnb1; slmap; vcl; myl9; fkbp1a; ltb4r; hsbp1; asph; tpm1; acta1; myl6; sgca; mylk*	9,99E-06
actomyosin based movement	11	*dysf; myh3; myl6b; myl9; tnnt2; myl4; myl2; acta1; myl6; myl1; actc1*	1,23E-04
negative regulation of cell proliferation	44	*tes; foxo4; msx2; dnajb2; dpt; gal; kmt2a; ptprf; rarg; fgfr3; cebpa; sst; nf1; cdh13; tgfb2; serpine2; timp2; rassf5; six5; bax; tnfrsf8; tgfb1i1; sfrp5; lefty2; gabbr1; myog; csk; pml; pla2g2a; hras; ddr1; rbm5; klf13; hrg; hyal1; osm; asph; il1rl1; ptk2b; dis3l2; speg; igf1; cdkn1c; col4a3*	2,43E-04
muscle organ development	17	*srpk3; foxo4; tagln3; myf5; des; utrn; myl4; myl1; zfhx3; sgce; myog; myh3; sgcb; itga7; speg; igf1; sgca*	3,91E-04
muscle hypertrophy	1	*igf1*	1,07E-03
smooth muscle cell migration	3	*plau; itgb3; ddr1*	2,07E-03
regulation of muscle filament sliding speed	2	*tnnt2; tnnc1*	2,38E-03
regulation of muscle contraction	7	*atp2a1; tnnt2; tnnc2; myl9; tpm1; tnnc1; casq2*	2,40E-03
skeletal muscle tissue development	10	*srpk3; zbtb18; myf5; myl6b; nf1; ctsb; tgfb2; myog; itga7; myl6*	2,51E-03
satellite cell maintenance involved in skeletal muscle regeneration	1	*igf1*	3,21E-03
myotube cell development	1	*igf1*	4,27E-03

cell differentiation	56	*clptm1; prmt7; znf281; metrn; foxo4; myf5; gli1; znf541; cebpa; amigo1; arhgap24; tcp11; bmpr1b; gadd45b; neurog1; serpine2; ifrd1; lgr4; hand1; ngrn; hmx3; zc3h12a; rps7; fgf3; uhrf2; pax5; piwil4; amh; dmrt1; ngfr; gadd45g; csrp2; ptk2b; creb3l4; neurod4; srpk3; rmdn3; ext2; fgfr3; elf3; insm1; camk2g; inha; angptl4; ndrg2; tgfb1i1; sfrp5; pura; myog; myrf; efnb3; jag2; wipf3; speg; rnf114; bmp8b*	4,64E-03
myoblast proliferation	1	*igf1*	5,34E-03
response to muscle activity involved in regulation of muscle adaptation	2	*sepn1; myog*	6,90E-03
adult somatic muscle development	2	*utrn; ifrd1*	6,90E-03
positive regulation of myoblast proliferation	1	*igf1*	8,53E-03
cardiac muscle contraction	8	*tnnt2; myl4; myl2; myl1; actc1; tpm1; tnnc1; casq2*	1,14E-02
positive regulation of cardiac muscle hypertrophy	1	*igf1*	1,28E-02
cardioblast differentiation	2	*tgfb2; ece2*	1,34E-02
muscle cell differentiation	6	*myf5; cdh15; ifrd1; abl1; myog; speg*	1,39E-02
regulation of cardiac muscle cell contraction	1	*casq2*	1,59E-02
skeletal muscle fiber development	5	*sepn1; ppp3ca; myog; acta1; homer1*	1,73E-02
positive regulation of cell differentiation	6	*fgfr3; ins; junb; bmpr1b; hsf4; neurod4*	2,35E-02
skeletal muscle cell differentiation	7	*atf3; myf5; lemd2; ankrd1; pax5; ngfr; foxn2*	2,42E-02
cardiac myofibril assembly	3	*myl2; pdgfra; actc1*	3,39E-02
muscle cell fate commitment	2	*myf5; myog*	4,24E-02
skeletal muscle thin filament assembly	2	*actc1; acta1*	4,24E-02
negative regulation of planar cell polarity pathway involved in cardiac muscle tissue morphogenesis	1	*lrp6*	4,88E-02
canonical Wnt signaling pathway involved in cardiac neural crest cell differentiation involved in heart development	1	*lrp6*	4,88E-02

regulation of smooth muscle cell-matrix adhesion	1	*plau*	4,88E-02
regulation of satellite cell activation involved in skeletal muscle regeneration	1	*gjd4*	4,88E-02
muscle cell fate specification	1	*myl2*	4,88E-02
myotome development	1	*wdr19*	4,88E-02
skeletal muscle organ development	1	*cntfr*	4,88E-02
muscle tissue development	1	*srpk3*	4,88E-02
striated muscle atrophy	1	*myog*	4,88E-02
regulation of myoblast fusion	1	*myog*	4,88E-02
