# Supplementary material for: Transcriptomic Profile of Primary Culture of Skeletal Muscle Cells Isolated from Semitendinosus Muscle of Beef and Dairy Bulls
Source: Int J Mol Sci. 2020 Jul 7;21(13):4794. doi: 10.3390/ijms21134794 (PMC7369917; doi:10.3390/ijms21134794)
Supplement: Supplementary file 1 [file ijms-21-04794-s001.zip › Table S3.pdf]

Table S3. Classification showing the involvement of genes common for LIM and HER breeds in selected biological processes associated with muscle development (*DAVID*).

Process	Number of genes	Gene symbol	FDR
muscle contraction	38	*aldoa; myl6; edn3; myl4; tnnc2; tnnc1; tacr2; myl1; utrn; pgam2; fkbp1a; ttn; tpm1; des; dysf; myl6b; ltb4r; ckmt2; slmap; asph; chrna1; actc1; acta1; myh3; myh2; gal; homer1; tnnt2; tnnt1; myh11; smpx; chrnb2; chrnb1; kbtbd10; chrne; casq2; sgca; chrng*	3,29E-07
muscle system process	39	*aldoa; myl6; edn3; myl4; tnnc2; tnnc1; tacr2; myl1; utrn; pgam2; fkbp1a; ttn; tpm1; des; dysf; myl6b; ltb4r; ckmt2; slmap; asph; chrna1; actc1; acta1; myh3; myh2; igf1; gal; homer1; tnnt2; tnnt1; myh11; smpx; chrnb2; chrnb1; kbtbd10; chrne; casq2; sgca; chrng*	1,49E-06
muscle organ development	43	*myl6; myl4; myl2; tnnc1; erbb3; des; myl1; utrn; eln; fkbp1a; foxo4; ttn; tpm1; tagln3; tgfb2; hand1; myl6b; gata6; speg; tdgf1; myog; ppp3ca; chrna1; col11a1; unc45b; srpk3; actc1; acta1; smad7; myh3; nf1; myf5; hspg2; igf1; csrp2; homer1; tnnt2; itga7; myh11; foxc2; sgce; sgca; sgcb*	3,71E-05
striated muscle contraction	17	*aldoa; myl4; actc1; tnnc2; tnnc1; myh3; myh2; myl1; pgam2; ttn; homer1; tpm1; tnnt1; smpx; chrna1; kbtbd10; casq2*	2,36E-04
muscle tissue development	28	*myl6; myl2; erbb3; tnnc1; eln; utrn; fkbp1a; ttn; tpm1; tgfb2; hand1; gata6; myl6b; tdgf1; myog; ppp3ca; chrna1; col11a1; actc1; acta1; smad7; nf1; myf5; hspg2; homer1; tnnt2; myh11; foxc2*	1,11E-03
striated muscle tissue development	27	*myl6; myl2; erbb3; tnnc1; eln; utrn; fkbp1a; ttn; tpm1; tgfb2; hand1; gata6; myl6b; tdgf1; myog; ppp3ca; chrna1; col11a1; actc1; acta1; smad7; myf5; hspg2; homer1; tnnt2; myh11; foxc2*	1,41E-03
extracellular matrix organization	24	*elf3; lum; nf1; myf5; eln; col3a1; cst3; hspg2; ccdc80; fkbp1a; spink5; aplp2; tgfb2; crispld2; col1a2; pdgfra; myh11; col12a1; foxc2; col1a1; lamc1; col11a1; smarca4; dpt*	4,87E-03
muscle tissue morphogenesis	11	*tnnt2; actc1; hand1; myl2; tnnc1; smad7; foxc2; fkbp1a; ttn; col11a1; tpm1*	2,95E-02
cardiac muscle tissue morphogenesis	11	*tnnt2; actc1; hand1; myl2; tnnc1; smad7; foxc2; fkbp1a; ttn; col11a1; tpm1*	2,95E-02

cardiac muscle tissue development	16	*actc1; myl2; smad7; tnnc1; hspg2; fkbp1a; ttn; tpm1; tgfb2; tnnt2; hand1; gata6; tdgf1; myh11; foxc2; col11a1*	2,96E-02
