# Supplementary material for: Transcriptomic Profile of Primary Culture of Skeletal Muscle Cells Isolated from Semitendinosus Muscle of Beef and Dairy Bulls
Source: Int J Mol Sci. 2020 Jul 7;21(13):4794. doi: 10.3390/ijms21134794 (PMC7369917; doi:10.3390/ijms21134794)
Supplement: Supplementary file 1 [file ijms-21-04794-s001.zip › Table S4.pdf]

Table S4. Primers used in qPCR.

Gene symbol	Accession number	Forward primer	Reverse primer	PCR product length
des	NM_001081575	5'AGAGGGGTTCTGAGGTCCATA3'	5'TTAGGAGGCAGTGAGGACAGA3'	169
erbb3	NM_001103105	5'ACGCCTGGCATCAGAATCATCG3'	5'ACCATTGACATCCTCTTCCTCTAACC3'	171
igf-1	XM_005206500	5'CAGAAGCAATGGGAAAAATCA3'	5'CGTGGGCTTGTTGAAATAAAA3'	242
myf5	NM_174116	5'ATGCCATCCGCTACATTGAG3'	5'ACGCTGTCAAAACTGCTGCT3'	178
myh3	NM_001101835	5'CTGGAGGAAATGAGGGATGA3'	5'CACTCTTGAGAAGGGGCTTG3'	211
myl2	NM_001035025	5'AGAATGACCTGAGGGACACC3'	5'ATCAGCCTTGAGCACTCCTT3'	215
myog	NM_001111325	5'GATAAAGCAAGGGACCAGAGTG3'	5'GGCATAGGAAGAGATGAACAGG3'	183
gapdh	NM_001034034	5'GTTTGTGATGGGCGTGAACC3'	5'GTCTTCTGGGTGGCAGTGAT3'	198
